# Cultural Humility Curriculum to Address Healthcare Disparities for Emergency Medicine Residents

**DOI:** 10.5811/westjem.2023.1.58366

**Published:** 2023-03-06

**Authors:** Ryan E. Tsuchida, Jessica Doan, Eve D. Losman, Adrianne N. Haggins, Robert D. Huang, Daniel J. Hekman, Marcia A. Perry

**Affiliations:** * University of Wisconsin, School of Medicine and Public Health, BerbeeWalsh Department of Emergency Medicine, Madison, Wisconsin; † University of Michigan Medical School, Department of Emergency Medicine, Ann Arbor, Michigan

## Abstract

**Introduction:**

Emergency medicine (EM) residency programs have variable approaches to educating residents on recognizing and managing healthcare disparities. We hypothesized that our curriculum with resident-presented lectures would increase residents’ sense of cultural humility and ability to identify vulnerable populations.

**Methods:**

At a single-site, four-year EM residency program with 16 residents per year, we designed a curriculum intervention from 2019–2021 where all second-year residents selected one healthcare disparity topic and gave a 15-minute presentation overviewing the disparity, describing local resources, and facilitating a group discussion. We conducted a prospective observational study to assess the impact of the curriculum by electronically surveying all current residents before and after the curriculum intervention. We measured attitudes on cultural humility and ability to identify healthcare disparities among a variety of patient characteristics (race, gender, weight, insurance, sexual orientation, language, ability, etc). Statistical comparisons of mean responses were calculated using the Mann-Whitney U test for ordinal data.

**Results:**

A total of 32 residents gave presentations that covered a broad range of vulnerable patient populations including those that identify as Black, migrant farm workers, transgender, and deaf. The overall survey response was 38/64 (59.4%) pre-intervention and 43/64 (67.2%) post-intervention. Improvements were seen in resident self-reported cultural humility as measured by their responsibility to learn (mean responses of 4.73 vs 4.17; *P* < 0.001) and responsibility to be aware of different cultures (mean responses of 4.89 vs 4.42; *P* < 0.001). Residents reported an increased awareness that patients are treated differently in the healthcare system based on their race (*P* < 0.001) and gender (*P* < 0.001). All other domains queried, although not statistically significant, demonstrated a similar trend.

**Conclusion:**

This study demonstrates increased resident willingness to engage in cultural humility and the feasibility of resident near-peer teaching on a breadth of vulnerable patient populations seen in their clinical environment. Future studies may query the impact this curriculum has on resident clinical decision-making.

## INTRODUCTION

The healthcare of vulnerable populations disproportionately falls to the emergency department (ED), which has become the safety net for many local communities.^1^ When patients access care through the ED, they often encounter emergency medicine (EM) trainees as a part of their care team. To provide equitable care it is important for EM trainees to understand that healthcare inequities and social determinants of health impact the diverse populations that they will encounter while working in the ED. While most agree that knowledge about cultural issues is important when providing clinical care, many trainees feel unprepared and unequipped to address the social needs of the populations they serve. 2

The Accreditation Council for Graduate Medical Education (ACGME) Common Program Requirements include trainee recognition and management of healthcare disparities through the domains of interpersonal and communication skills, systems-based practice, and quality improvement.^3^ The ACGME’s 2018 Clinical Learning Environment Report (CLER) highlighted that “across most clinical learning environments, formal education and training on cultural competency did not address the specific populations served by the institution.”^4^ Additionally, the report noted that programs with a healthcare disparities curriculum focused on generic experiences and did not address the specific populations served by the physicians in those institutions.

Despite this call to action, there is little information about how to help trainees recognize the breadth of disparities that they encounter at the bedside.^5^ Anecdotally, healthcare disparities in the medical education curriculum are taught as long-form lectures with PowerPoint presentations, typically with an expert as the teacher. This passive approach comes with challenges including lack of learner engagement and difficulty achieving desired educational objectives and outcomes.^6^ Alternate strategies include community-based efforts, simulation, and case-based learning. However, these approaches are time- and resource-intensive and therefore not possible for many training programs.

The approach to addressing health disparities and social determinants of health in medical training programs has largely focused on teaching cultural competency. While cultural competency focuses on delivering quality care to patients with diverse beliefs, attitudes, values, and behaviors it has also been criticized as being one dimensional, promoting finite knowledge, and having a discrete endpoint.^7^–^9^ The framework of cultural humility is an alternative approach. As defined by Tervalon and Murray-Gargia, cultural humility is “a lifelong commitment to self-evaluation and self-critique, to redressing the power imbalances in the patient-physician dynamic, and to developing mutually beneficial and non-paternalistic clinical and advocacy partnerships with communities on behalf of individuals and defined populations.”^10^ Cultural humility emphasizes a growth mindset with a lifelong dynamic process of self-reflection.

**Population Health Research Capsule**

What do we already know about this issue?

*Emergency medicine residency programs have variable approaches to educating residents on recognizing and managing healthcare disparities*.

What was the research question?

*Can residents identify vulnerable patient populations and use cultural humility in a resident-led lecture to address healthcare disparities?*

What was the major finding of the study?

*Residents demonstrated increased cultural humility (P < 0.001) and awareness of patient bias due to race and gender (P <0.001)*.

How does this improve population health?

*The long-term desired outcome is for residents to address biases in healthcare delivery and reduce disparities through equitable patient care*.

Previous studies describing cultural humility curricula with family medicine residents, pediatric residents, physical therapy students, and medical students have shown positive results.^11^–^14^ As described in those studies, cultural humility is taught through instructor-led presentations, and cases are drawn from simulation, patient panels, or home visits. Our study introduces a novel healthcare disparities curriculum based on resident-led presentations, drawn from their own clinical encounters, that encourage the practice of self-directed learning and cultural humility. Our first hypothesis was that a resident-led lecture series that sought to address patients’ social needs within their local community would increase residents’ appreciation for cultural humility. Our second hypothesis was that residents are capable of identifying patient populations that experience healthcare disparities from the community that they serve in their ED.

## METHODS

### Study Design

This prospective observational study from July 1, 2019–June 30, 2021 examines the impact of a curriculum intervention on EM residents’ appreciation for cultural humility and attitudes toward healthcare disparities over two academic years by administering a pre- and post-intervention, self-reported survey. This study was deemed exempt by the University of Michigan IRB (HUM 00164660).

### Population

Participants in this study were EM residents in a single, four-year EM residency program with 16 residents per year. These residents rotate at three core training sites: a tertiary care academic ED; a small city community ED; and an urban county ED. At this program, EM residency didactics are held once weekly. All residents are required to attend at least 70% of the sessions.

### Curricular Design

We used Kern’s six-step model for medical education curriculum development.^15^ We used the ACGME CLER report and annual program review as our general needs assessment. A specific-needs assessment electronic survey was deployed to current residents to identify specific knowledge and skills gaps. We identified four barriers to asking patients about their social needs: 1) fear of threatening the doctor/patient relationship; 2) lack of knowledge of the resources available to patients; 3) lack of knowledge of the community they serve; and 4) limited time with the patient in an ED encounter.

Following this initial survey, we designed a novel longitudinal curriculum integrated into the existing weekly EM residency didactic structure. We proposed a case-based, near-peer teaching curriculum (ie, learner as teacher) and centered our curriculum on junior residents as content developers and presenters. As part of our intervention, in the spring each rising second-year resident was required to sign up to give a 15-minute presentation on healthcare disparities in the upcoming academic year. A total of 16 15-minute lectures were scheduled for each year.

Prior to the start of each academic year, rising second-year residents were given a document outlining the background and objectives for the lecture series (Appendix 1). The learner-teachers were asked to 1) briefly describe a patient encounter where observed inequities challenged the statement, “Quality care is equitable care”; 2) describe how to increase awareness of patients at risk for disparate care; and 3) provide actionable information on at least one institutional, community, or state resource that could be used to address the observed barrier. During their presentation, residents were expected to provide a brief overview of the disparity and available local resources, and to conclude with a facilitated group discussion. A running list of previous lecture topics was provided. While repeating a similar topic was not prohibited, residents were instructed to focus on a unique intersectional perspective to avoid duplication.

From July 2019–February 2020, all presentations were given in person. Like all other resident didactics, the format was switched to an online virtual format in March 2020 as a result of the coronavirus 2019 pandemic. The lectures were temporally spaced to allow integration of healthcare disparities topics into the broader curriculum and to avoid isolating these talks on a specific day. We felt it was important to emphasize that education on healthcare disparities had equal importance to education on clinical and scientific topics within the field of EM. During the first year of implementation, residents were scheduled to present on different weeks. To smooth the scheduling demands, the following year the lectures were scheduled in pairs.

### Assessment

We assessed the impact of the curriculum via a pre- (June 2019) and post- (June 2021) online survey tool (Qualtrics XM, Provo, UT) that measured residents’ attitudes on cultural humility and their ability to identify healthcare disparities among a variety of patient characteristics (race, gender, weight, insurance, sexual orientation, language, ability, etc) (Appendix 2). All 64 current residents at each time point — at the time of study implementation and at the conclusion of the assessment — were invited to complete the surveys. To maintain respondents’ anonymity, we did not collect their demographics.

To maximize internal validity and minimize self-report bias, we created the survey by combining questions from two previously validated and published studies that were then reviewed by a group of EM medical education experts prior to survey administration.^16^,^17^ Questions were selected by study authors with content expertise to reflect the aims of the study hypothesis. One set of questions was used to measure their cultural humility by asking residents about their cultural awareness, attitudes, and behaviors using a five-item Likert scale. Another set of questions asked residents about their perceptions of the differences in care patients received in the ED based on their identities, using a four-item scale of 0–25% of the time through 75–100% of the time. Lastly, in the post-implementation survey, additional questions were included for formal evaluation and assessment of the curriculum and to allow for narrative feedback. We reviewed this feedback for themes and have included representative narratives in the discussion.

### Analysis

We performed statistical comparisons of mean responses using Student’s *t*-test, and did comparisons between pre- and post-intervention survey response distributions using the Mann-Whitney U test (also known as the Wilcoxon rank-sum test) for unpaired ordinal data. All data cleaning and statistical analysis was done using R (The R Foundation for Statistical Computing, Indianapolis, IN). 18 We followed the Strengthening the Reporting of Observational Studies in Epidemiology (STROBE) guidelines for this observational study.^19^

## RESULTS

From 2019 to 2021, 32/32 (100%) second-year residents presented on 28 unique healthcare disparities topics covering a broad range of vulnerable patient populations (Table 1). During the two-year study period, 16/24 (66%) months had at least one resident presentation scheduled for the lecture series. The overall survey response rate was 38/64 (59.4%) pre-intervention and 43/64 (67.2%) post-intervention (Table 2). Responses were obtained from residents at all levels of training.

**Figure 1:**
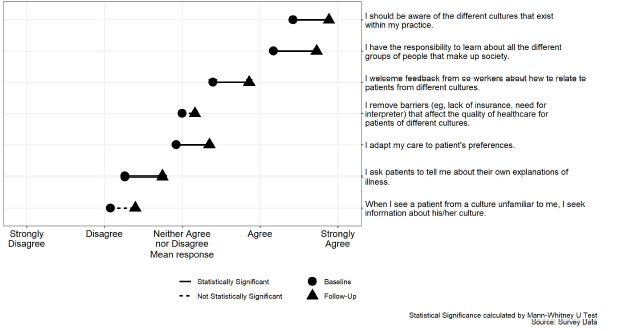
Measurement of cultural humility pre- and post- implementation responses.

**Figure 2:**
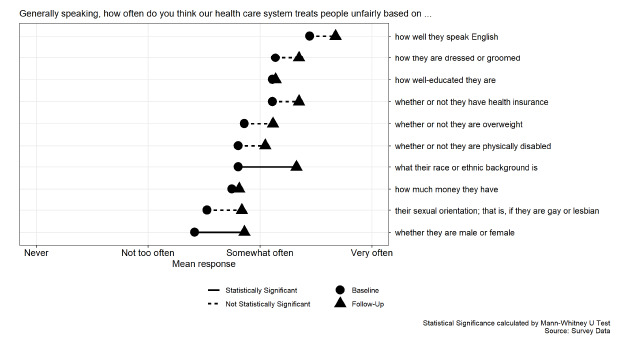
Rate of recognizing healthcare disparities pre- and post- implementation responses.

Questions about cultural humility, specifically cultural attitudes and behavior, that had higher rates of self-reported behavior following the curriculum intervention include “I ask patients to tell me about their own explanations of illness” (*P*=0.030); “I adapt my care to patient’s preferences” (*P*=0.030); “I welcome feedback from co-workers about how to relate to patients from different cultures” (*P*=0.009); “I have the responsibility to learn about all the different groups of people that make up society” (*P*<0.001); and “I should be aware of the different cultures that exist within my practice” (*P*<0.001) (Figure 1). Residents reported a statistically significant increase in concern that patients are treated differently in the healthcare system based on their race (*P* < 0.001) and gender (*P* < 0.001) (Figure 2). The remaining survey questions, although not statistically significant at the 5% level, trended in a similar direction (Appendix 3).

At the end of the study period, 38 of 42 residents (90.5%) reported that the lecture series had changed their approach to caring for patient populations who are marginalized, 30 (71.4%) reported increased knowledge with regard to caring for patient populations who are marginalized, 30 (71.4%) reported increased awareness of their current knowledge gaps in caring for patient populations who are marginalized, and 26 (54.2%) reported an increased desire to learn more about caring for patient who are marginalized.

We also obtained qualitative feedback regarding the curriculum design, and representative comments are included below.

### Representative Positive Comments

“It was great to see so many different topics presented. Each presentation included literature or resources that I wasn’t previously aware of.”

“I felt like I learned pertinent information from these lectures, and it made me proud of my program for actively teaching about these topics.”

“I learned a lot from my classmates.”

“I am glad this was added into conference.”

“[It was] helpful to illuminate ongoing disparities in a multitude of areas and domains.”

### Representative Critical Comments

Some of the lectures definitely could have used more polish and have been better prepared ahead of time, I think lecture quality undermined some of the points - a Zoom lecture has to be fantastic to grab and hold attention; otherwise it gets ignored.

“As many of the higher yield topics are presented, [it is] harder to come up with a good topic.”

“Changing the lecture series to a different format (sim/community outreach) could also be interesting.”

“I think it would benefit from… few larger lectures, rather than 16, 15-minute lectures [per year].”

## DISCUSSION

In this two-year longitudinal didactic curriculum, second-year EM residents at a four-year academic EM program led self-reflective discussions on healthcare disparities to engage peers on patient encounters in their clinical learning environment. As compared to pre-intervention, residents reported an increased desire to learn about patients at risk for healthcare disparities and a change in their approach to improve care for patients marginalized in the healthcare system. This finding suggests an increase in residents’ sense of cultural humility, as the lectures spurred their interest to address knowledge gaps related to these patients. Residents identified a wide range of topics and were able to identify many unique cases where patients were marginalized by the healthcare system.

Importantly, these topics were identified by residents without specific topic selection a priori. We noted a correlation between the curriculum intervention and resident recognition of racial and gender disparities experienced by their patients. A similar increase in recognizing disparities was seen among all historically marginalized groups queried. The statistical differences noted for racial and gender disparities may have been due to their relative frequency in the clinical context. Additionally, these identities may be more readily apparent in clinical encounters compared to an individual’s income, level of education, or sexual orientation.

We designed and implemented a unique curriculum that encourages residents to use the fundamentals of cultural humility, rather than cultural competency, to promote learner-directed didactics and introspection. There is a consistent trend away from cultural competency and toward cultural humility.^7^,^8^ Lekas et al emphasizes that training in cultural competency risks stereotyping, stigmatizing, and “othering” of patients and offers little acknowledgment of the intersectionality of multiple marginalized identities. The authors argue that physicians should instead be trained in cultural humility, which focuses on self-reflection, is more patient-centered, addresses a physician’s openness to share power with the patient, and emphasizes the goal of learning continuously from their patients.

Anger et al discusses the theoretical differences between cultural humility and cultural competency and underscores the value of shifting to cultural humility. Uniquely, the emphasis on self-reflection in cultural humility facilitates learners to explore their unconscious and conscious biases. Recently, the Association of American Medical Colleges released competencies on diversity, equity, and inclusion that specifically include assessing the practice of cultural humility.^20^ Additionally, the ACGME has begun to explore the incorporation of cultural humility into residency education as evidenced by the creation of the Pursuing Excellence Health Care Disparities Collaborative*.21* The goals of this initiative include cultural humility, social determinants of health, and quality improvement.

One study surveying EM residency program directors found that approximately two-thirds of responding programs had cultural competency as part of their curriculum.^22^ Similar to what was reported by the ACGME, over 90% of these curricula used generic structured didactics with a focus on race and ethnic disparities. Those authors identified notable gaps in incorporating additional healthcare disparities such as limited English proficiency, gender identity and sexual orientation, and social determinants of health. In a recent study by Ward-Gaines et al, EM residents were exposed to various health equity topics using simulation immersion.^23^ Residents reported a greater understanding of various healthcare disparities. While their study described cultural competency outcomes, the authors discussed the importance of self-reflection – a key tenet of cultural humility.

Our study is the first to show how an EM residency can incorporate cultural humility into its didactic curriculum. One important outcome of our curriculum is that residents are exposed to a wide range of topics not limited to race and ethnicity. Residents selected patient populations with disparities defined by social isolation, immigration, incarceration, sexual orientation, language, deafness, and mental health. We believe that this cultural humility-based healthcare disparities curriculum in EM residency programs is a feasible approach that can be implemented into existing didactic structures.

An important feature of our curriculum is the focus on cultural humility, specifically self-reflection and lifelong learning. Residents were encouraged to select clinical encounters where a social determinant of health was a potential barrier to care. They presented the clinical case, and ways to overcome the barriers, to their peers and faculty in a flipped classroom style — with the learner as a teacher. Importantly, the case-based model encouraged critical self-reflection as trainees were asked to share real-life episodes of unequal care encountered during their clinical shifts. In addition, they were tasked with discovering and sharing local resources that could be brought to the bedside to address patients’ needs in the ED and upon discharge (eg, how to get a peer-recovery coach to come to the ED to counsel and support a post-overdose patient; how to access the local food pantries; how to ask about pronouns when caring for transgender individuals; what local advocacy groups support youth in crisis; etc). After participating in the curriculum, residents reported increased awareness of and concern for individuals marginalized within the healthcare system. While the statistical significance varied in each domain, the trend of increasing concern over time was consistent. In addition, resident responses also revealed increased awareness of their knowledge gaps and a desire to learn more about populations that are marginalized. This is consistent with the goal of cultural humility as a lifelong and dynamic process.

Collectively, these are important skills for emergency physicians to have throughout their career. Emergency physicians may work in various practice settings and are exposed to innumerable cultural customs and changing patient demographics. It is not feasible to achieve a “competency” that is individualized to every patient. An emphasis of learning from the individual patient and self-reflection provides a unique advantage of cultural humility over cultural competency. Future studies may assess this impact through measuring encounter-level outcomes such as resource utilization, connection to community resources, or ED return visits. We have adapted our own healthcare disparities curriculum to encourage more engagement with ED-based operational metrics as stratified by various patient demographics.^24^

## LIMITATIONS

This was an observational study without a control group to assess the impact that time had over the two-year study period. Statistical analysis was limited by a small sample size precluding any subset analysis by residency cohort. Individual-level impact was not assessed as respondent identifiers were not recorded. Changes in behavior were self-reported, and we did not assess change in care delivery. It is possible that some differences in responses of our pre- and post-implementation survey were due to increased awareness of healthcare disparities from COVID-19 and the increased recognition of structural racism in the United States that was highlighted by the disproportionate incidence of mortality in Black patients.^25^

Additionally, our curriculum was designed and initially implemented roughly nine months before the regional impact of COVID-19 required that all educational content to be converted from in person to a virtual format. Anecdotally, the switch to virtual format led to a tendency for more time to be filled with presentations, which left less time available for discussion. We anticipate that had this transition not occurred, the curriculum would have had a greater impact.

## CONCLUSION

This resident-driven lecture series empowered learners to identify and present on healthcare disparities relevant to their clinical learning environment. Over the study period, residents were encouraged to engage as lifelong learners. Residents demonstrated growth in cultural humility through self-reflection and lifelong learning, and they gained a greater appreciation for existing healthcare disparities. We believe future curricula should reinforce a longitudinal, integrated approach, and attempt to assess curriculum impact on direct patient care.

## Supplementary Information







## Figures and Tables

**Table 1:** Lecture titles. Second-year residents presented 32 lectures between 2019–2021, covering 28 unique topics.

Advocating for Incarcerated Populations	Healthcare Disparities in Athletes
Alcohol Use	Health Literacy
Alcohol Use Disorder *	Housing Insecurity
Amish Healthcare	Identifying Sex Trafficking in the Emergency Department
Care of Patients with Sickle Cell Disease	Immigrant and Latino Healthcare/Border Medicine
Caring for Incarcerated Patients *	Mental Health and Minorities
Coronavirus Disease 19 Healthcare Disparities	Migrant Farmworkers
Coronavirus Disease 19-Related Inequities *	Non-English Speaking Patients and Interpreters
Culture Differences in Pain Expression and Emergency Department Pain Management	Patient requesting Clinician Based on Bias
Deaf/Hard-of-hearing Health Challenges in the Time of Coronavirus Disease 19	Patients Boarding with Inpatient Psychiatric Needs
Disparities in Clinical Trials	Race and Pain Management
Disparities in Psychiatric Care	Rural Health Disparities
Disparities in Trauma	Social Isolation
Financial Barriers	Transgender Care
Food Insecurity	Transgender Health *
Healthcare Disparities Among Refugee Populations	Transportation

* Repeated topics.

**Table 2:** Resident survey response rates by postgraduate training year during each phase of the study

	Overall	Pre-intervention survey	Post-intervention survey
n	84	40	44
First year (Intern)	27 (32.5)	14 (35.0)	13 (30.2)
Second year	20 (24.1)	12 (30.0)	8 (18.6)
Third year	22 (26.5)	11 (27.5)	11 (25.6)
Fourth year	14 (16.9)	3 (7.5)	11 (25.6)
