# Supplementary material for: Cultural Humility Curriculum to Address Healthcare Disparities for Emergency Medicine Residents
Source: West J Emerg Med. 2023 Mar 6;24(2):119–26. doi: 10.5811/westjem.2023.1.58366 (PMC10047734; doi:10.5811/westjem.2023.1.58366)
Supplement: Supplementary file 1 [file wjem-24-119-s001.docx]

**EM2 – Healthcare Disparities Case Presentation**

Previous Clinical Learning Environment Review feedback of residency programs identified knowledge deficits amongst trainees related to healthcare disparities:

*“Across most clinical learning environments, education and training on health care disparities and cultural competency was largely generic, and often did not address the specific populations served by the institution…… residents and fellows reported that learning about health care disparities and cultural competency was happening in an ad-hoc manner.”*

In order to address this gap in education, and ultimately provide equitable care, we must create deliberate opportunities to educate trainees on social determinants of health, identify at-risk patients, available resources, and ways to apply this knowledge to their practice to reduce health care disparities.

To address healthcare disparities in emergency medicine, you will be asked to give a 15-minute case presentation of a case encounter (from observations or direct clinical encounters at any of the 3 training sites), where social determinants of health was a barrier to delivering high quality health care. Social determinates of health include:


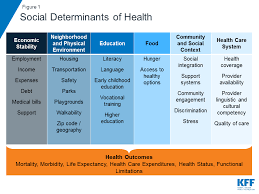


Case Objectives:

1. Describe a patient encounter where observed inequities impaired the following statement: “Quality Care is Equitable Care.”
2. Increase awareness of patients at risk for disparate care.
3. Provide background a single accessible institutional, community, or state resource that can be used to address the observed barrier.

If a resource cannot be identified, propose a potential intervention/solution.

Your presentation should be brief but thoughtful. We recommend preparing a presentation roughly 10 minutes in length to allow for a group discussion. Encourage the audience to suggest instances when they’ve encountered a similar situation and how they advocated for equitable care.

*[Redacted for blinded review]* has a commitment to reducing health and healthcare disparities and has signed American Hospital Association (AHA) #123 for Equity Pledge which aims to reduce institutional disparities. This activity aims to improve your residency “education on identifying and reducing healthcare disparities relevant to the patient population served by the clinical site” uncovered by Accreditation Council for Graduate Medical Education/Clinical Learning Environment Review (ACGME /CLER).

Residents and Faculty you may reach out to for guidance:

- *[redacted for blinded review]*
