# Supplementary material for: Cultural Humility Curriculum to Address Healthcare Disparities for Emergency Medicine Residents
Source: West J Emerg Med. 2023 Mar 6;24(2):119–26. doi: 10.5811/westjem.2023.1.58366 (PMC10047734; doi:10.5811/westjem.2023.1.58366)
Supplement: Supplementary file 2 [file wjem-24-119-s002.docx]

Emergency Medicine Residents Perspectives on Health Care Disparities - Follow Up

Start of Block: Block 1

Q18 This is a follow-up survey to assess the impact of the health care disparities curriculum. Our goal is to use this information to inform future directions of the curriculum. **This survey is ideally completed on a computer and should take about 5 minutes to complete.** Your participation is voluntary and responses are confidential. Thank you! [redacted for blinded review] IRB exempt.

Q17 What is your current your year of training in Emergency Medicine?

- First year (intern) (1)
- Second year (2)
- Third year (3)
- Fourth year (4)

| Page Break |  |
| --- | --- |

Please indicate the degree that you agree or disagree with the following statements.

Q1 I have the responsibility to learn about all the different groups of people that make up society.

- Strongly disagree (1)
- Somewhat disagree (2)
- Neither agree nor disagree (3)
- Somewhat agree (4)
- Strongly agree (5)

Q2 I should be aware of the different cultures that exist within my practice.

- Strongly disagree (1)
- Somewhat disagree (2)
- Neither agree nor disagree (3)
- Somewhat agree (4)
- Strongly agree (5)

| Page Break |  |
| --- | --- |

Q9 Please indicate the percentage of time you perform the following actions.

Q3 When I see a patient from a culture unfamiliar to me, I seek information about his/her culture.

- 0-25% (1)
- 26-50% (2)
- 51-75% (3)
- 76-100% (4)

Q10 I ask patients to tell me about their own explanations of illness.

- 0-25% (1)
- 26-50% (2)
- 51-75% (3)
- 76-100% (4)

Q11 I welcome feedback from co-workers about how to relate to patients from different cultures.

- 0-25% (1)
- 26-50% (2)
- 51-75% (3)
- 76-100% (4)

Q12 I adapt my care to patient’s preferences.

- 0-25% (1)
- 26-50% (2)
- 51-75% (3)
- 76-100% (4)

Q14
I remove barriers (e.g. lack of insurance, need for interpreter) that affect the quality of
healthcare for patients of different cultures.

- 0-25% (1)
- 26-50% (2)
- 51-75% (3)
- 76-100% (4)

| Page Break |  |
| --- | --- |

Q16
Generally speaking, how often do you think our health care system treats people unfairly
based on the following?

|  | Very Often (1) | Somewhat Often (2) | Not too often (3) | Never (4) |
| --- | --- | --- | --- | --- |
| what their race or ethnic background is (1) |  |  |  |  |
| whether or not they have health insurance (2) |  |  |  |  |
| whether they are male or female (3) |  |  |  |  |
| how well they speak English (4) |  |  |  |  |
| whether or not they are physically disabled (5) |  |  |  |  |
| how they are dressed or groomed (6) |  |  |  |  |
| how well-educated they are (7) |  |  |  |  |
| whether or not they are overweight (8) |  |  |  |  |
| how much money they have (9) |  |  |  |  |
| their sexual orientation—that is, if they are gay or lesbian (10) |  |  |  |  |

| Page Break |  |
| --- | --- |

Q23 Please rate how much you agree with the following:


The health care disparities lecture series increased my knowledge in caring for patients of marginalized populations.

- Strongly agree (4)
- Somewhat agree (5)
- Neither agree nor disagree (6)
- Somewhat disagree (7)
- Strongly disagree (8)

Q24 Because of the health care disparities lecture series, I am more aware of my current knowledge gaps in caring for patients of marginalized populations.

- Strongly agree (4)
- Somewhat agree (5)
- Neither agree nor disagree (6)
- Somewhat disagree (7)
- Strongly disagree (8)

Q26 The health care disparities lecture series increased my desire to learn more about caring for patients of marginalized populations.

- Strongly agree (4)
- Somewhat agree (5)
- Neither agree nor disagree (6)
- Somewhat disagree (7)
- Strongly disagree (8)

Q22 How often has the health care disparities lecture series changed your approach to caring for patients of marginalized populations?

- Always (17)
- Most of the time (18)
- About half the time (19)
- Sometimes (20)
- Never (21)

| Page Break |  |
| --- | --- |

Q19 Please share your experience VIEWING the health care disparities lecture series.

________________________________________________________________

________________________________________________________________

________________________________________________________________

________________________________________________________________

________________________________________________________________

| Page Break |  |
| --- | --- |

Display This Question:

If What is your current your year of training in Emergency Medicine? = Second year

Or What is your current your year of training in Emergency Medicine? = Third year

Q20 Please share your experience PRESENTING your health care disparities lecture. Please describe any barriers or challenges.

________________________________________________________________

________________________________________________________________

________________________________________________________________

________________________________________________________________

________________________________________________________________

| Page Break |  |
| --- | --- |

Q21 Please provide feedback on how you would like to see the lecture series improved.

________________________________________________________________

________________________________________________________________

________________________________________________________________

________________________________________________________________

________________________________________________________________

End of Block: Block 1
