# Supplementary material for: Cultural Humility Curriculum to Address Healthcare Disparities for Emergency Medicine Residents
Source: West J Emerg Med. 2023 Mar 6;24(2):119–26. doi: 10.5811/westjem.2023.1.58366 (PMC10047734; doi:10.5811/westjem.2023.1.58366)
Supplement: Supplementary file 3 [file wjem-24-119-s003.docx]

*Attitudes on cultural humility (1 = strongly disagree and 5 = strongly agree) and Awareness of health care disparities (where 1 = never and 4 = very often). SD = Standard Deviation. IQR = Interquartile Range*

|  |  | | **Pre-Intervention Survey** | | | | **Post-Intervention Survey** | | | |  |  |
| --- | --- | --- | --- | --- | --- | --- | --- | --- | --- | --- | --- | --- |
| **Question Wording** | **Response Options** | | **Mean (SD)** | | **Median (IQR)** | | **Mean (SD)** | | **Median (IQR)** | | **W-Statistics (Wilcoxon Test)** | **P-value** |
| What is your current your year of training in Emergency Medicine? | 4 (First Year - Fourth Year) | | 2.08 (0.97) | | 2 (2) | | 2.47 (1.18) | | 3 (2.5) | | 699 | 0.129 |
| I have the responsibility to learn about all the different groups of people that make up society. | 5 (Strongly Disagree - Strongly Agree | | 4.17 (0.96) | | 4 (1) | | 4.73 (0.5) | | 5 (0.2) | | 555.5 | 0.001 |
| I should be aware of the different cultures that exist within my practice. | 5 (Strongly Disagree - Strongly Agree | | 4.42 (0.96) | | 5 (1) | | 4.89 (0.39) | | 5 (0) | | 609 | 0.001 |
| When I see a patient from a culture unfamiliar to me, I seek information about his/her culture. | 5 (Strongly Disagree - Strongly Agree | | 2.08 (0.88) | | 2 (2) | | 2.4 (0.88) | | 2 (1) | | 672 | 0.149 |
| I ask patients to tell me about their own explanations of illness. | 5 (Strongly Disagree - Strongly Agree | | 2.26 (0.98) | | 2 (1.8) | | 2.74 (0.98) | | 3 (1) | | 596 | 0.030 |
| I welcome feedback from co-workers about how to relate to patients from different cultures. | 5 (Strongly Disagree - Strongly Agree | | 3.39 (0.92) | | 4 (1) | | 3.86 (0.35) | | 4 (0) | | 609 | 0.009 |
| I adapt my care to patient's preferences. | 5 (Strongly Disagree - Strongly Agree | | 2.92 (0.9) | | 3 (2) | | 3.35 (0.61) | | 3 (1) | | 620.5 | 0.030 |
| I remove barriers (e.g. lack of insurance, need for interpreter) that affect the quality of healthcare for patients of different cultures. | 5 (Strongly Disagree - Strongly Agree | | 3 (0.79) | | 3 (0.5) | | 3.16 (0.69) | | 3 (1) | | 759 | 0.420 |
| Generally speaking, how often do you think our health care system treats people unfairly based on ... what their race or ethnic background is | 4 (Never - Very Often) | | 2.81 (0.82) | | 3 (1) | | 3.33 (0.64) | | 3 (1) | | 501 | 0.004 |
| Generally speaking, how often do you think our health care system treats people unfairly based on ... whether or not they have health insurance | 4 (Never - Very Often) | | 3.11 (0.85) | | 3 (1.2) | | 3.35 (0.75) | | 4 (1) | | 658 | 0.220 |
| Generally speaking, how often do you think our health care system treats people unfairly based on ... whether they are male or female | 4 (Never - Very Often) | | 2.42 (0.65) | | 2 (1) | | 2.86 (0.71) | | 3 (1) | | 532.5 | 0.010 |
| Generally speaking, how often do you think our health care system treats people unfairly based on ... how well they speak English | 4 (Never - Very Often) | | 3.44 (0.73) | | 4 (1) | | 3.67 (0.52) | | 4 (1) | | 652 | 0.157 |
| Generally speaking, how often do you think our health care system treats people unfairly based on ... whether or not they are physically disabled | 4 (Never - Very Often) | | 2.81 (0.82) | | 3 (1) | | 3.05 (0.79) | | 3 (1) | | 651.5 | 0.197 |
| Generally speaking, how often do you think our health care system treats people unfairly based on ... how they are dressed or groomed | 4 (Never - Very Often) | | 3.14 (0.8) | | 3 (1) | | 3.35 (0.65) | | 3 (1) | | 671.5 | 0.272 |
| Generally speaking, how often do you think our health care system treats people unfairly based on ... how well-educated they are | 4 (Never - Very Often) | | 3.11 (0.82) | | 3 (1) | | 3.14 (0.56) | | 3 (0) | | 785.5 | 0.904 |
| Generally speaking, how often do you think our health care system treats people unfairly based on ... whether or not they are overweight | 4 (Never - Very Often) | | 2.86 (0.9) | | 3 (2) | | 3.12 (0.66) | | 3 (1) | | 655.5 | 0.211 |
| Generally speaking, how often do you think our health care system treats people unfairly based on ... how much money they have | | 4 (Never - Very Often) | | 2.75 (0.84) | | 3 (1) | | 2.81 (0.82) | | 3 (1) | 739.5 | 0.722 |
| Generally speaking, how often do you think our health care system treats people unfairly based on ... their sexual orientation; that is, if they are gay or lesbian | | 4 (Never - Very Often) | | 2.53 (0.84) | | 2 (1) | | 2.84 (0.72) | | 3 (1) | 615 | 0.094 |
